# Aging-related trajectories of lung function in the general population—The Doetinchem Cohort Study

**DOI:** 10.1371/journal.pone.0197250

**Published:** 2018-05-16

**Authors:** Sandra H. van Oostrom, Peter M. Engelfriet, W. M. Monique Verschuren, Maarten Schipper, Inge M. Wouters, Marike Boezen, Henriëtte A. Smit, Huib A. M. Kerstjens, H. Susan J. Picavet

**Affiliations:** 1 Center for Nutrition, Prevention and Health Services, National Institute for Public Health and the Environment, Bilthoven, the Netherlands; 2 Division of Environmental Epidemiology, Institute for Risk Assessment Sciences (IRAS), Utrecht University, Utrecht, The Netherlands; 3 Department of Epidemiology, UMCG, Groningen, the Netherlands; 4 Julius Center for Health Sciences and Primary Care, University Medical Center Utrecht, Utrecht, the Netherlands; 5 Department of Pulmonary Diseases, UMCG, Groningen, the Netherlands; Ospedale S. Corona, ITALY

## Abstract

The objective of this study was to explore trajectories of lung function decline with age in the general population, and to study the effect of sociodemographic and life style related risk factors, in particular smoking and BMI. For this purpose, we used data from the Doetinchem Cohort Study (DCS) of men and women, selected randomly from the general population and aged 20–59 years at inclusion in 1987–1991, and followed until the present. Participants in the DCS are assessed every five years. Spirometry has been performed as part of this assessment from 1994 onwards. Participants were included in this study if spirometric measurement of FEV_1_, which in this study was the main parameter of interest, was acceptable and reproducible on at least one measurement round, leading to the inclusion of 5727 individuals (3008 females). Statistical analysis revealed three typical trajectories. The majority of participants followed a trajectory that closely adhered to the Global Lung Initiative Reference values (94.9% of men and 96.4% of women). Two other trajectories showed a more pronounced decline. Smoking and the presence of respiratory complaints were the best predictors of a trajectory with stronger decline. A greater BMI over the follow-up period was associated with a more unfavorable FEV_1_ course both in men (β = -0.027 (SD = 0.002); P < 0.001) and in women (β = -0.008 (SD = 0.001); P < 0.001). Smokers at baseline who quit the habit during follow-up, showed smaller decline in FEV_1_ in comparison to persistent smokers, independent of BMI change (In men β = -0.074 (SD = 0.020); P < 0.001. In women β = -0.277 (SD = 0.068); P < 0.001). In conclusion, three typical trajectories of age-related FEV1 decline could be distinguished. Change in the lifestyle related risk factors, BMI and smoking, significantly impact aging-related decline of lung function. Identifying deviant trajectories may help in early recognition of those at risk of a diagnosis of lung disease later in life.

## Introduction

Chronic respiratory diseases rank high as cause of morbidity and mortality worldwide.[1, 2] As age is the most important risk factor for COPD, besides smoking, the disease burden due to chronic respiratory disease is likely to increase, especially in countries with aging populations.

Much of the morbidity from chronic lung disease is due to failing lung function. Decline of lung function often progresses insidiously and once symptoms become manifest, the accumulated damage has become irreversible. Measurement of lung function by means of spirometry has therefore become a mainstay in the diagnosis of chronic lung disease and in the monitoring of treatment effect. As lung function declines with aging, the effect of age needs to be taken into account.[3, 4] Indeed, understanding the impact of age on the development of airflow limitation is considered a research priority in current respiratory medicine.[5] Existing spirometric reference values have been derived from cross-sectional studies of healthy individuals of various ages [6, 7]. However, in order to gain a more accurate insight into the change of lung function over the life course, longitudinal studies with sufficiently long follow up of spirometric parameters are indispensable. In particular, such studies offer a clearer view on interindividual variation in lung function trajectories, which might help in distinguishing pathological decline from ‘normal’ aging.

The study we present analyzed lung function change during aging in the general population. We used data from the population-based Doetinchem Cohort Study[8] of men and women aged 20 to 59 years at baseline (1987–1991). The data included spirometric measurements, performed four times in a row, at five-year intervals. We aimed to identify typical aging-related trajectories of lung function as measured by forced expiratory volume in one second (FEV_1_).[9, 10] The hypothesis was that ‘latent’ heterogeneity of lung function in the population can be revealed by statistical analysis, using a powerful ‘data-driven’ method. After having characterized a number of distinct trajectories, we investigated to what extent baseline sociodemographic and lifestyle characteristics determine the likelihood of following a particular trajectory. We also studied the effect of changes in BMI and of quitting with smoking on the course of lung function during follow-up.

## Materials and methods

### Study population

The study design of the Doetinchem Cohort study has been described elsewhere.[8] Participants were selected randomly by age- and sex-stratified sampling from the civil registries of Doetinchem, a small town in the Netherlands. Inclusion started in the period 1987–1991. Almost all participants are white, and ethnically native. From the participants in the first measurement round (n = 12,405, participation rate 62%), a random sample of 7,768 were invited for a second measurement round (1993–1997). This last random sample was invited again in 1998–2002 (round 3), 2003–2007 (round 4), and 2008–2012 (round 5). The response rates for all follow-up measurements varied between 75% and 80%, resulting in 6113, 4916, 4520, and 4017 participants for rounds 2, 3, 4, and 5, respectively. Lung function measurements were included from the first half of 1994 onwards. Therefore, for the present analyses data from the period 1994 to 2012 were used and round 2 was considered as baseline.

The study was conducted according to the principles of the World Medical Association Declaration of Helsinki and its amendments since 1964, and in accordance with the Medical Research Involving Human Subject Act (WMO). The protocols for subsequent rounds were approved by the Medical Ethical Committee (*Medisch Ethische Commissie*) of TNO (rounds 2 and 3), respectively the Medical Ethical Committee (*Medisch-Ethische Toetsingscommissie*) of University Medical Center Utrecht (rounds 4 and 5).

All participants gave written informed consent.

### Spirometry

Lung function (without bronchodilation) was measured by trained paramedics using a heated pneumotachometer (E Jaeger, Wurzburg, Germany), with the examined person in a seated upright position. FEV_1_ was recorded as the greatest FEV_1_ of ≥3 technically acceptable measurements (out of a maximum of 8 trials), with the requirement that the highest and second highest value matched within 5% (Quality grade A as described in Enright *et al*.[11]). Participants were included in the analyses if their FEV_1_ was acceptable and reproducible on at least one measurement round (N = 5727: 2719 males and 3008 females). For participants who were included in the analyses, measurements on other rounds that were not acceptable or non-reproducible, were excluded (i.e. considered as ‘missing’). We excluded 904 examinations that did not meet the quality requirements. In addition, pregnant women were excluded for the round that took place during their pregnancy (n = 65).

### Sociodemographic, lifestyle and respiratory health characteristics

Measured height and body weight were used to calculate body mass index, which was used as a continuous measure, or categorized as normal (BMI lower than 25 kg/m^2^), overweight (BMI ≥ 25 and < 30 kg/m^2^), and obese (BMI 30 kg/m^2^ or above). Education was categorized into three levels (low, moderate, and high). Work status was defined as having a paying job or not, and household composition as living alone or not. Smoking status was categorized as current smoker, ex-smoker, and never-smoker. Also numbers of pack-years at baseline were estimated. Physical activity was assessed with a self-administered questionnaire designed for the international European Prospective Investigation Into Cancer and Nutrition study, to which a question was added on sports and other strenuous leisure-time physical activities.[12] Being physically active were considered those who spent at least 3.5 hours on moderate-to-vigorous leisure-time physical activities and heavy work, in conformity with international guidelines.[13] Questionnaire assessment of COPD and asthma symptoms was based on the Dutch component of the European Community Respiratory Health Survey.[14] COPD symptoms were: chronic (occurring on most days for at least 3 months a year) cough, chronic sputum production or breathlessness. Breathlessness was defined as shortness of breath when walking on level ground with people of the same age. Asthma symptoms were wheezing in the past 12 months, shortness of breath at night in the past 12 months, or a self-reported physician’s diagnosis of asthma. All participants were asked whether they used medication for respiratory symptoms in the preceding 24 hours.

### Statistical analyses

Our primary aim was to model within-person change of FEV_1_ as observed at four different time points. We used latent class mixture modeling (LCMM) that allows identifying a number of ‘typical’ trajectories in order to verify the hypothesis that the population is made up of heterogeneous subgroups, making as few *a priori* assumptions as seemed reasonable. That is, first we derived a best fitting model (see further below), separately for men and women, using the complete dataset and including only age (centered and scaled) as the independent variable, adjusting for body length (centered and scaled).[15]

We briefly summarize how we arrived at the best-fitting models for men (N = 2719) and women (N = 3008).

For each latent class the mean trajectory of FEV_1_ was modeled as a smooth function of age at examination and length. The deviation of individuals from the mean class trajectories was modeled by the addition of random intercepts and slopes of age. A ‘best’ model was selected by optimizing over different smoothness parameters, numbers of classes, and ‘link functions’. As criterion for optimization we used the Bayesian Information Criterion (BIC). Uncertainty was incorporated by estimating the individuals’ (posterior) probability of membership for each of the identified trajectories. Mean predicted FEV_1_ values over the life course were plotted for each trajectory (assigning individuals to the class with highest posterior probability). Curves were truncated to avoid extrapolation beyond the observations. Similar curves were plotted of FVC trajectories for the same classes as were identified based on the FEV_1_ analyses.

All LCMM analyses were done using statistical software R and the package lcmm.[16, 17]

To compare our longitudinal trajectories with spirometric reference values we also graphed in the figures the FEV_1_ and FVC reference values, using the equations developed by the Global Lung Initiative (GLI) (http://www.lungfunction.org, accessed 1 July 2016).

#### Determinants of class (trajectory) membership

After having determined the optimum number of classes and growth parameters, we assigned each individual to the class for which posterior probability was highest, resulting in a distribution over classes. Differences between classes for a number of baseline characteristics (sociodemographic and lifestyle characteristics) were tabulated. In addition, differences in baseline FEV_1_, and absolute and relative decline in FEV_1_ were reported.

Next, the influence of baseline socio-demographic and lifestyle characteristics on trajectory probability was explored using multivariable weighted multinomial logistic regression. Assigned FEV_1_ trajectory membership was taken as the dependent variable, weighted for the maximum posterior probability over the trajectories. The trajectory to which the highest number of subjects were assigned was taken as the reference category.

All of these latter analyses were performed in SAS version 9.3 (SAS Institute, Cary, NC, USA).

#### BMI change and smoking behavior during follow-up

In order to study the influence of potentially modifiable life style related risk factors on FEV_1_ decline, we assessed the effect of change during follow-up in smoking behavior (smoking cessation) and in BMI on FEV_1_. The effect of BMI change was evaluated by incorporating BMI as time-varying variable in the LCMM model. That is, the value of BMI at each consecutive round, corresponding to the age of the participant at that particular investigation, was entered into the model. The relation between BMI and FEV_1_ was adjusted for several variables, apart from age and length, including baseline FVC.

In order to have sufficient power to detect an effect of smoking cessation, we analyzed a reduced dataset consisting of individuals who smoked at baseline, using mixed linear modelling (R package lme4), in which we compared persistent smokers with quitters. Smoking cessation was defined based on smoking status at each round. If there was a change in smoking status from ‘current smoker’ to ‘former smoker’ from one round to the next, and this changed status persisted at subsequent rounds, this was taken to signify that this participant had quit smoking during follow up. For adjustment, the following baseline variables were included in the model: length, exposure to passive smoking, number of pack-years, COPD-like symptoms, and asthma-like symptoms.

*P*-values smaller than 0.05 were considered statistically significant. Hypothesis tests were 2-sided.

## Results

Table 1 shows baseline characteristics. The mean age was 46 years, with range 26 to 65 years. Almost one third of men and women were current smokers. Mean FEV_1_ was 4.0 L for men and 3.0 L for women.

**Table 1:** Baseline sociodemographic, respiratory health, and lifestyle characteristics of men and women in the Doetinchem Cohort Study.

	Men	Women
	N = 2719	N = 3008
Age in years (mean (SD))	46.6 (9.9)	46.1 (10.0)
Age categories		
26–34 yr (N (%))	391 (14	462 (15)
35–44 yr	831 (31)	985 (33)
45–54 yr	885 (33)	894 (30)
55–66 yr	612 (23)	667 (22)
Educational level		
Low (%)	1060 (39)	1681 (56)
Medium	932 (34)	759 (25)
Height in cms (mean (SD))	179.0 (6.7)	166.1 (6.3)
Job (N (%))	2084 (79)	1352 (47)
Living alone (N (%))	158 (7)	212 (8)
FEV_1_ in Liters (mean (SD))	4.0 (0.8)	3.0 (0.5)
FVC in Liters (mean (SD)	5.3 (1.0)	3.9 (0.6)
FEV_1_/FVC	0.76 (0.08)	0.78 (0.07)
COPD symptoms (N (%))	339 (12)	346 (12)
Asthma symptoms (N (%))	356 (13)	387 (13)
Respiratory medication in 24 hrs preceding spirometry (N (%))	30 (1)	36 (1)
BMI in kg/m^2^ (mean (SD))	25.8 (3.1)	25.2 (4.2)
Overweight (N (%))	1300 (48)	1015 (34)
Obese	248 (9)	346 (12)
Smoker (N (%))	844 (31)	907 (30)
Ex-smoker	1140 (42)	1036 (34)
Physically active (N (%))	1291 (56)	1405 (56)

### Trajectories

All measurements meeting quality requirements were included in modeling trajectories. Numbers of participants with 1, 2, 3, or 4 valid measurements were: 451 (16.6%), 428(15.8%), 765 (28.1%) and 1075 (39.5%), for men, and 555 (18.4%), 464 (15.4%), 811 (27.0%), and 1178 (39.2%) for women. In the first year of round 2 (1993) spirometry was not included, implying that 423 men and 475 women had a maximum of 3 available FEV_1_ measurements.

**Fig 1:**
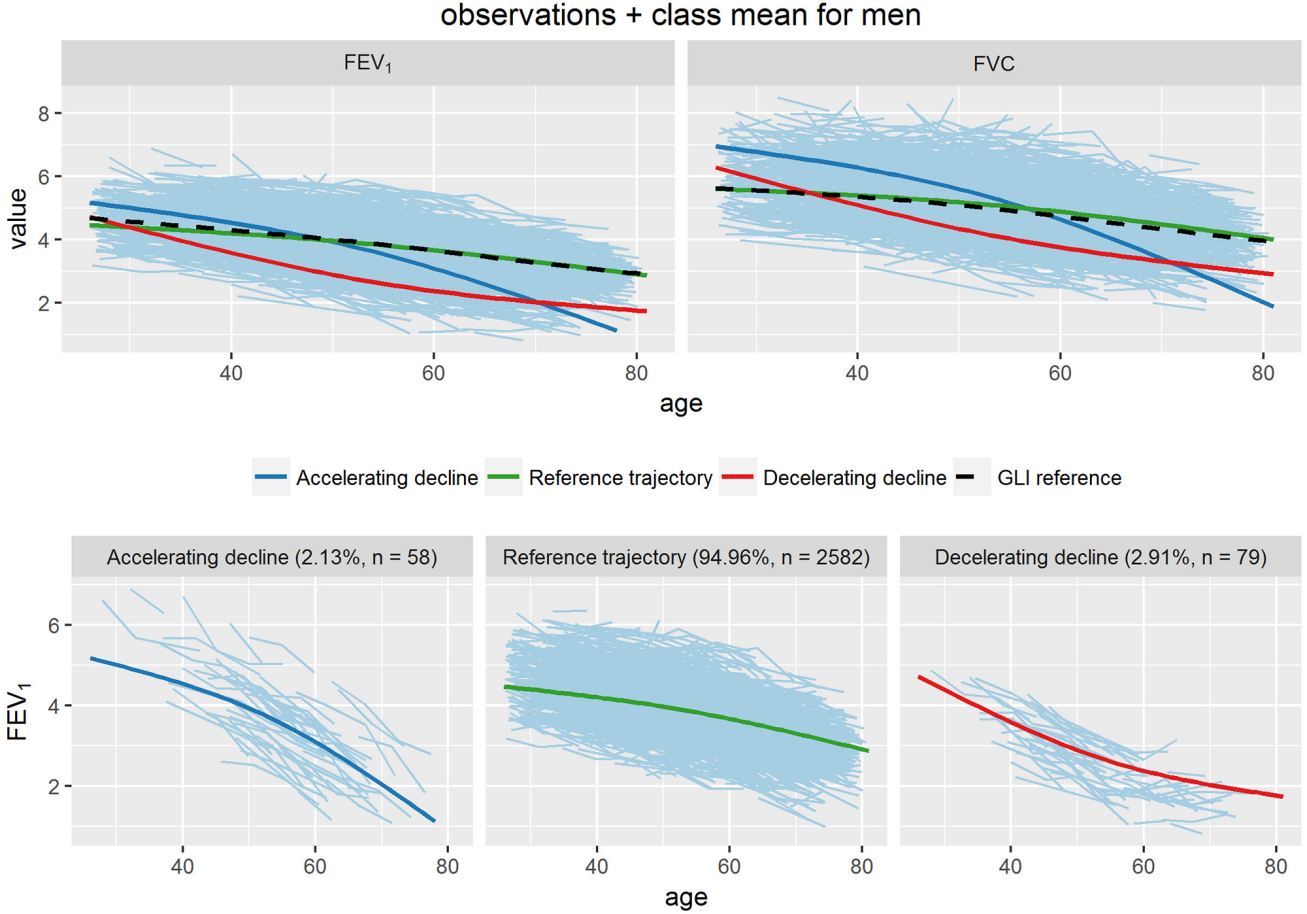
Trajectories of FEV_1_ (Liters) for men. The curves in the upper left panels of the figure represent the ‘average’ FEV_1_ trajectory for the individuals in each group, after classification into groups based on the greatest probability of class membership. The upper right panels show the FVC trajectories for these groups. The bottom panels display the individual FEV_1_ curves of the members of each group separately.

**Fig 2:**
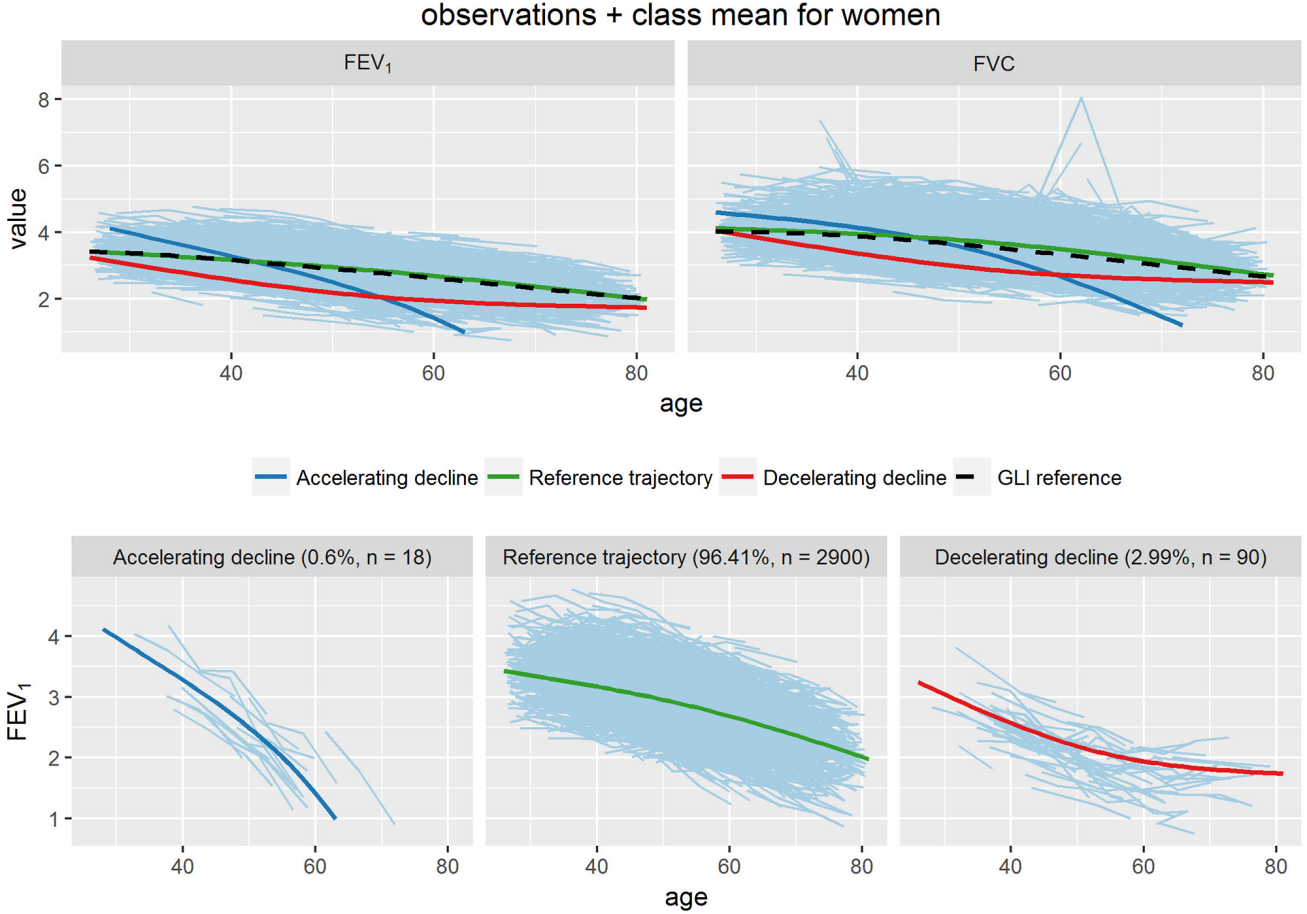
Trajectories of FEV_1_ (Liters) for women.

With these measurements as input, latent class mixture modelling identified three distinct trajectories both in men and in women. These are shown in Figs 1 and 2. Also shown are the FVC trajectories for the same groups, as well as the individual FEV_1_ curves of the members of each group separately.

The majority of participants followed a FEV_1_ trajectory that closely adhered to the Global Lung Initiative Reference values (95.0% of men and 96.4% of women), characterized by steady moderate decline from an age of approximately 30 years onwards (upper left panels Figs 1 and 2). We labeled this the ‘reference’ trajectory. Two other trajectories could be distinguished with a more pronounced decline. One ‘accelerating decline’ trajectory had a relatively high initial level followed by a rate of decline that increased with age (2.1% of men; 0.6% of women). Another ‘decelerating decline’ trajectory showed an initial level not far from the reference level, followed by a relatively strong initial decline returning to more moderate rates with increasing age (3.0% of men; 3.0% of women). The FVC curves show very similar shapes of the trajectories for the three groups (upper right panels in Figs 1 and 2).

### Characteristics of trajectory groups

Mean (SD) baseline FEV_1_ and FVC values for the three male and female trajectories, as well as the mean Z-scores, are shown in Table 2. The table also displays absolute and relative changes in FEV_1_ and FVC per group.

**Table 2:** Baseline FEV_1_, absolute and relative change in FEV_1_ for men and women in each of the FEV_1_ trajectories.

**Men**	**Trajectories according to rate of decline**		
	**Decelerating decline**	**Reference trajectory**	**Accelerating decline**
Baseline FEV_1_			
mean (SD), L	4.0 (1.2)	4.0 (0.7)	2.8 (1.0)
Z score (mean)	-0.11	-0.22	-2.43
Absolute change in FEV_1_ (mL/yr)^a^	-111.4 (36.1)	-31.1 (28.6)	-59.5 (42.4)
Relative change in FEV_1_ (%/yr)^a^	-3.0 (1.5)	-0.8 (0.8)	-1.9 (1.6)
Baseline FVC			
mean (SD), L	5.5 (1.2)	5.2 (0.9	4.5 (1.1))
Z score (mean)	0.61	0.08	-1.09
Absolute change in FVC (mL/yr)^a^	-115.4 (59.9)	32.9 (45.5)	-60.9 (38.7))
Relative change in FVC (%/yr)^a^	2.2 (1.3)	-0.6 (1.3)	1.3 (0.9)
**Women**	**Trajectories according to rate of decline**		
	**Decelerating decline**	**Reference trajectory**	**Accelerating decline**
Baseline FEV_1_			
mean (SD), L	2.2 (0.5)	3.0 (0.5)	2.8 (0.7)
Z score (mean)	-2.19	-0.01	-0.77
Absolute change in FEV_1_ (mL/yr)^a^	-32.3 (30.9)	-25.9 (21.0)	-97.7 (23.4)
Relative change in FEV_1_ (%/yr)^a^	-1.3 (1.5)	-0.9 (0.8)	-3.5 (1.2)
Baseline FVC			
mean (SD), L	3.2 (0.6)	3.8 (0.6)	3.8 (0.8)
Z score (mean)	-1.14	0.21	-0.21
Absolute change in FVC (mL/yr)^a^	-32.3 (29.0)	-27.6 (34.9)	-75.2 (37.9)
Relative change in FVC (%/yr)^a^	-1.0 (0.9)	0.7 (0.9)	-2.1 (1.3)

^a^ Absolute and relative change in FEV_1_ are determined over the longest available period, for most respondents a period of 15 years.

Men reporting asthma (2.42 (1.17 5.02)) or COPD symptoms (2.34 (1.13 4.81)) at baseline were more likely to be in the ‘accelerated decline’ group than those not reporting such symptoms (Table 3), as were smokers (3.29 (1.06 10.19)).

**Table 3:** Baseline sociodemographic and lifestyle determinants of the trajectories for men compared to the most common FEV_1_ trajectory (reference trajectory).

Men	Trajectories according to rate of decline		
	Decelerating decline	Reference trajectory	Accelerating decline
Educational level			
Low	0.48 (0.20 1.18)	REF	1.65 (0.61 4.43)
Medium	1.08 (0.49 2.37)	REF	2.05 (0.77 5.50)
High	-	REF	-
No paid job	1.08 (0.46 2.56)	REF	0.97 (0.44 2.14)
Living alone	0.92 (0.26 3.28)	REF	1.11 (0.38 3.25)
COPD symptoms	2.14 (0.94 4.84)	REF	**2.34 (1.13 4.81)**
Asthma symptoms	1.62 (0.70 3.74)	REF	**2.42 (1.17 5.02)**
BMI			
Normal	-	REF	-
Overweight	1.68 (0.82 3.45)	REF	1.34 (0.67 2.69)
Obese	1.48 (0.47 4.66)	REF	1.83 (0.73 4.62)
Smoking			
Smoker	2.23 (0.81 6.14)	REF	**3.29 (1.06 10.19)**
Ex-smoker	1.42 (0.57 3.58)	REF	2.17 (0.72 6.56)
Never-smoker	-	REF	-
Tobacco exposure at home/work	1.15 (0.52 2.55)	REF	1.66 (0.72 3.85)
Physically inactive	1.05 (0.55 2.00)	REF	1.08 (0.58 2.01)

The table presents odds ratios and 95% confidence intervals. Odds ratios are reported as obtained in the multivariable model. In addition, all odds ratios were adjusted for age, length at baseline, and the use of respiratory medication.

The most conspicuous group differences at baseline for women were also mainly related to smoking, and the presence of respiratory symptoms: smokers had a greater risk of being in the ‘accelerating decline’ group (10.98 (1.22 98.49)) or in the ‘decelerating decline’ group (3.17 (1.33 7.56)), compared to non-smokers. Those reporting COPD or asthma symptoms were more likely to be in the ‘decelerating decline’ group than those not reporting respiratory symptoms. (Table 4).

**Table 4:** Baseline sociodemographic and lifestyle determinants of the trajectories for women compared to the most common FEV_1_ trajectory (reference trajectory).

Women	Trajectories according to rate of decline		
	Decelerating decline	Reference trajectory	Accelerating decline
Educational level			
Low	1.98 (0.74 5.29)	REF	4.95 (0.37 67.17)
Medium	1.98 (0.70 5.57)	REF	3.11 (0.20 47.85)
High	-	REF	-
No paid job	1.28 (0.70 2.34)	REF	0.58 (0.16 2.02)
Living alone	1.17 (0.49 2.78)	REF	0.78 (0.07 8.15)
COPD symptoms	**3.29 (1.78 6.10)**	REF	1.43 (0.31 6.58)
Asthma symptoms	**2.23 (1.18 4.24)**	REF	3.16 (0.80 12.47)
BMI			
Normal	-	REF	-
Overweight	0.73 (0.38 1.39)	REF	1.27 (0.34 4.83)
Obese	0.96 (0.41 2.25)	REF	1.32 (0.19 9.16)
Smoking			
Smoker	**3.17 (1.33 7.56)**	REF	**10.98 (1.22 98.49)**
Ex-smoker	1.00 (0.40 2.50)	REF	1.09 (0.11 10.52)
Never-smoker	-	REF	-
Tobacco exposure at home/work	1.92 (0.83 4.43)	REF	0.49 (0.08 3.08)
Physically inactive	1.21 (0.70 2.09)	REF	0.39 (0.10 1.52)

The table presents odds ratios and 95% confidence intervals. Odds ratios are reported as obtained in the multivariable model. In addition, all odds ratios were adjusted for age, length at baseline, and the use of respiratory medication.

### The effect of BMI change during follow-up and smoking cessation on the course of FEV_1_ decline

The effect of these lifestyle related risk factors was studied on a reduced dataset, due to missing values in the added variables. The effect of BMI change during follow-up was studied in the three-class model derived on the full dataset, but excluding observations with missing values in one or more of the included covariates. As missingness was selective, with smokers and those with respiratory symptoms at baseline being more likely to be excluded due to missing values (S1 Table), this reduced data set of 2084 men and 2260 women therefore is not entirely representative of the full dataset.

#### BMI change

Table 5 displays the effect of BMI change over the follow up period on FEV_1_, and of the baseline covariates that were included for adjustment. Two multivariable models were compared, one including an interaction term between BMI and baseline FVC and one without.

**Table 5:** The effects of BMI change during follow-up, adjusted for baseline variables.

Variables	Coefficients (SE)	p-value
**Men**		
*Model 1*: *Interaction FVC x BMI not included*		
Smoking at baseline	-0.095 (0.031)	0.002
Pack-years at baseline	-0.001 (0.001)	0.293
Passive smoking	-0.011 (0.018)	0.566
COPD symptoms at baseline	-0.011 (0.028)	0.711
Asthma symptoms at baseline	-0.159 (0.027)	< 0.001
Baseline FVC	0.546 (0.0123)	< 0.001
**BMI**	**-0.027 (0.002)**	**< 0.001**
*Model 2*: *Interaction FVC x BMI included*		
Smoking at baseline	-0.085 (0.031)	0.001
Pack-years at baseline	-0.002 (0.001)	0.172
Passive smoking	-0.012 (0.018)	0.535
COPD symptoms at baseline	-0.011 (0.029)	0.691
Asthma symptoms at baseline	-0.159 (0.027)	< 0.001
Baseline FVC	0.804 (0.052)	< 0.001
**BMI**	**-0.023 (0.010)**	**0.022**
BMI x baseline FVC	-0.00970 (0.002)	< 0.001
**Women**		
*Model 1*: *Interaction FVC x BMI not included*		
Smoking at baseline	-0.052 (0.021)	0.012
Pack-years at baseline	-0.004 (0.001)	< 0.001
Passive smoking	-0.001 (0.013)	0.929
COPD symptoms at baseline	-0.026 (0.019)	0.180
Asthma symptoms at baseline	-0.078 (0.017)	< 0.001
Baseline FVC	0.488 (0.011)	< 0.001
**BMI**	**-0.008(0.001)**	**< 0.001**
*Model 2*: *Interaction FVC x BMI included*		
Smoking at baseline	-0.055 (0.021)	0.009
Pack-years at baseline	-0.004 (0.001)	< 0.001
Passive smoking	-0.003 (0.013)	0.823
COPD symptoms at baseline	-0.029 (0.019)	0.134
Asthma symptoms at baseline	-0.080 (0.017)	< 0.001
Baseline FVC	0.659 (0.039)	< 0.001
**BMI**	**0.016 (0.005)**	**0.003**
BMI x baseline FVC	-0.007 (0.001)	< 0.001

The table displays the estimated effects of varying BMI on FEV_1_ during follow-up. In addition to the covariates shown, the models were adjusted for age and length. Due to missing values for some variables, the numbers of subjects included were substantially lower than in the original model (male: N = 2084; female: N = 2260)

Greater BMI during follow-up was significantly associated with stronger FEV_1_ decline (P < 0.001, both in men and in women, model 1). The models also show that baseline FVC is strongly correlated with FEV_1_ levels during follow-up. Both in men and women there was a highly significant interaction between BMI and baseline FVC: the negative effect of BMI on FEV_1_ increases with larger values of FVC on baseline.

#### Smoking cessation

The dataset used to assess the effect of smoking cessation consisted of 492 men, all smokers at baseline, of whom 184 stopped smoking during follow-up and 308 persisted with the habit, and 525 women (201 versus 324). Table 6 shows the estimated effect of smoking cessation and the variables included in the model for adjustment. Smoking cessation was associated with a highly significantly greater FEV_1_ in comparison with persistent smoking, both in men and in women, independent of BMI. In women, the positive effect of smoking cessation was lesser at greater BMI’s, as shown by a significant negative interaction between the two.

**Table 6:** The effects of smoking cessation during follow-up, adjusted for baseline variables and for BMI.

Variables*	Coefficients (95% CI)	p-value
**Men**		
Pack-years at baseline	-0.008 (0.002)	< 0.001
COPD symptoms at baseline	-0.210 (0.061)	0.001
Asthma symptoms at baseline	-0.104 (0.067)	0.119
Baseline length	0.040 (0.004)	< 0.001
BMI	-0.027 (0.004)	< 0.001
Quit smoking during follow-up	0.074 (0.020)	< 0.001
**Women**		
Pack-years at baseline	-0.010 (0.002)	< 0.001
COPD symptoms at baseline	-0.035 (0.049)	0.474
Asthma symptoms at baseline	-0.124 (0.048)	0.011
Baseline length	0.030 (0.003)	< 0.001
BMI	-0.003 (0.002)	0.146
Quit smoking during follow-up	0.277 (0.068)	< 0.001
Interaction smoking cessation x BMI*	-0.008 (0.003)	0.001

*In men the interaction was not significant, and therefore not included in the model.

The table displays the estimated effects for 492 men, 184 quitters versus 308 persistent smokers, and 525 women, 201 quitters versus 324 persistent smokers. The variables displayed were adjusted for age (spline coefficients not shown)

## Discussion

The findings of this study confirm that change of lung function with age in the vast majority of adults follows a course that closely adheres to (GLI) reference values. Two deviant trajectories marked by increased rates of decline are seen in a minority of cases. In one of these trajectories, the rate of decline seems to accelerate with increasing age, whereas in the second there appears to be a return to a more moderate rate at older ages. Baseline determinants of the likelihood of following an unfavorable trajectory were the presence of respiratory complaints and smoking. Smoking was especially a predictor of a deviant course in women. Increases in BMI during follow-up were associated with stronger FEV_1_ decline, both in men and women. Smokers who persisted smoking showed a greater decline than those who quit smoking during follow-up.

The hypothesis that spirometric parameters of individuals in a population sample may follow distinct trajectories of decline, depending on the presence of risk factors, was proposed by Fletcher and Peto in 1977. This notion has since then been explored in several population-based studies. In most of these, the aging-related lung function change was analyzed with the aim of revealing different courses in subgroups of individuals defined by a prespecified criterion, such as smoking status.[18–22] In this study, we used methods of statistical clustering analysis to ascertain the existence of subgroups in lung function change in the general population without *a priori* classification of individuals on the basis of risk factors. Although admittedly exploratory and experimental, this approach is in line with the increasing recognition that chronic lung disease has a heterogeneous pathogenesis.[18, 23–25] Considering that chronic lung disease develops gradually over time, distinguishing distinct patterns (trajectories) in the evolvement of lung function with age could help in gaining more insight into the various phenotypes of chronic lung disease.[23] Different trajectories may result from ‘normal’ aging mechanisms complicated by the development of pathologic processes.[26–29] Imaging studies, for instance, have shown that pathological patterns are present in a substantial proportion of asymptomatic individuals.[30–33]

Even ‘normal’ rates of lung function decline may lead to COPD.[34] This shows that a trajectory reflects the life course as a whole.[35] FEV_1_ is determined by the maximally attained level in early adulthood, the age at onset of decline, and the (also age-dependent) rate of decline [36]; it is influenced by genetics, lifestyle and environmental exposures.[37, 38]

As the initial cohort was a random sample from the ‘healthy’ population, it is not surprising that the vast majority followed a course (our reference trajectory) largely in line with that of the GLI reference values as a function of age. These reference values, or predicted values given sex, height, ethnicity and age, were derived from cross-sectional studies.[6, 7, 39] In several earlier studies, discrepancies were noted when average decline with age was estimated from cross-sectional data compared to longitudinal data.[20, 40–43] These discrepancies have been attributed to cohort or period effects, or to ‘attrition’ bias. A recent large scale study, however, found no indication for secular trends.[44] Our study confirms the absence of substantial cohort effects (data not shown).

Almost 5% of the participants followed one of two trajectories characterized by a stronger rate of decline, and thus may be at increased risk of a diagnosis of airflow limitation, or COPD, at some point in life. Especially the trajectory with accelerating decline, is likely to be associated with an increased risk of future overt airflow limitation.[33, 45, 46] However, as those with missing values were more likely to have more unfavorable risk profiles (S1 Table), we might have underestimated the number of participants having trajectories with a stronger decline.

Baseline factors associated with the probability of a trajectory of increased decline were, not surprisingly, being a smoker and having respiratory symptoms. Being a current smoker is the most well-known risk factor for a low FEV_1_ as well as an accelerated decline. Also BMI is a modifiable risk factor for poor lung function.[47] Negative correlations have been reported between BMI and several spirometric parameters, including FEV_1_ and FVC, both in cross-sectional and in longitudinal studies.[48–51] Of note, FVC seems to be more affected than FEV_1_, with the result that the FEV_1_/FVC ratio might even increase, which would be interpreted as an absence of ‘obstructive’ airflow limitation.

We did not find a significantly higher risk for adults who were obese at baseline for a poor FEV_1_ trajectory over the life course. However, we did find that a higher BMI over the follow-up period was significantly associated with a stronger decline in FEV_1_, while baseline FVC was positively correlated with FEV_1_. In addition, there was a strong interaction between BMI change and baseline FVC in their effect on FEV_1_. We interpret this as an indication that the effect of BMI on FEV_1_ is largely mediated via a negative impact on FVC, but more research is needed to further disentangle this relation.

An important advantage of the long follow-up of this study was the ability to assess the benefits of quitting with smoking. Those who stopped smoking during follow-up ended up with higher FEV_1_ values than those who persisted in the habit. This finding corroborates the results of several other studies.[19, 42, 52]) As smoking cessation often leads to weight gain and this, in turn, may partly offset the positive effect of quitting smoking, we included BMI in our model.[53] The beneficial effects of smoking we found are thus adjusted for possible changes in BMI. In women, the positive effects of smoking cessation appeared to be reduced at greater BMI’s.

The practical relevance of gaining insight into these trajectories is the potential ability to recognize an ‘at risk’ pattern at an early stage, which, in turn, would allow early intervention. Moreover, the identified trajectories account for ‘hidden heterogeneity’, which may help in developing better prediction models. Although it is unlikely that spirometric screening in the general population would ever be a feasible or cost-effective approach, screening of individuals fulfilling a risk profile, for instance in general practice, could result in important health benefits. [54, 55]

### Strengths and limitations

The data for this study came from a long-running population-based study, providing insight into the evolution of lung function over the life course. Particular strengths of this study are the prospective data collection, the long duration of the follow-up, the high participation rates, and the consistent methodology applied for the spirometry measurements. We further applied relatively novel and powerful software, in exploring a ‘data-driven’ approach.[10] However, applying statistical methods of clustering analysis to longitudinal data also has its limitations. The selection of the optimal model is not always straightforward. There is no consensus on definite criteria for determining the number of classes or subgroups. Furthermore, the approach assumes a priori that distinct developmental trajectories in lung function exist.[9] Our findings of the existence of three distinct trajectories therefore will certainly need to be validated in other cohort studies. Also, in order to be able to study more in detail the determinants of the trajectories and the potential for prediction and interventions, larger data sets, for instance created by combining existing ones, are needed.

A further limitation inherent in most prospective cohort studies is selective attrition, in this case a greater propensity of more respiratory healthy participants to remain in the study during extended follow-ups. Those who were completely lost to follow-up, were excluded from our analyses. Moreover, in studying the effects of covariates on the course of lung function during follow-up, those with missing values in the covariates had to be excluded. In addition, the lack of ethnic subgroups in the cohort might be considered a limitation.

## Conclusion

This is the first time group-based trajectory modelling was applied to explore age-related trajectories in FEV_1_ in the general population. Future studies in large prospective population-based cohorts should confirm the existence of these trajectories, and the utility of distinguishing a number of (pheno)typical trajectories in early recognition of those at increased risk of developing chronic lung disease.

## Supporting information

S1 Table Baseline characteristics of individuals with missing data. Comparison of observed characteristics between participants with complete and incomplete data for the baseline covariates included in the model.
(DOCX)
