# Supplementary material for: Aging-related trajectories of lung function in the general population—The Doetinchem Cohort Study
Source: PLoS One. 2018 May 16;13(5):e0197250. doi: 10.1371/journal.pone.0197250 (PMC5955530; doi:10.1371/journal.pone.0197250)
Supplement: S1 Table — Comparison of observed characteristics between participants with complete and incomplete data for the baseline covariates included in the model. (DOCX) [file pone.0197250.s001.docx]

**Baseline characteristics of individuals with missing data:** comparison of observed characteristics between participants with complete and incomplete data for the baseline covariates included in the model.

|  | **Men with complete data**  **N=1897** | **Men with incomplete data**  **N=822** | **P** | **Women with complete data**  **N=2056** | **Women with incomplete data**  **N=952** | **P** |
| --- | --- | --- | --- | --- | --- | --- |
| Age (mean (sd)) | 45.8 (9.7) | 46.8 (10.4) | 0.02 | 45.0 (9.7) | 47.1 (10.6) | <0.01 |
| Length (mean (sd)) | 179.3 (6.5) | 178.2 (7.2) | <0.01 | 166.3 (6.2) | 165.4 (6.5) | <0.01 |
| Low educational level (N (%)) | 640 (34) | 420 (51) | <0.01 | 1042 (51) | 639 (67) | <0.01 |
| Job (N (%)) | 1521 (82) | 563 (71) | <0.01 | 996 (50) | 356 (39) | <0.01 |
| Living alone (N (%)) | 1468 (6) | 70 (10) | <0.01 | 128 (8) | 84 (10) | <0.01 |
| COPD symptoms (N (%)) | 217 (11) | 122 (15) | 0.01 | 220 (11) | 126 (13) | <0.01 |
| Asthma symptoms (N (%)) | 218 (12) | 138 (17) | <0.01 | 258 (13) | 129 (14) | 0.45 |
| Respiratory medication in 24 hrs preceding spirometry (N (%)) | 22 (1) | 8 (1) | 0.54 | 24 (1) | 12 (1) | 0.95 |
| BMI (mean (sd)) | 25.7 (3.0) | 26.0 (3.4) | <0.01 | 25.0 (4.0) | 25.7 (4.4) | <0.01 |
| Smoker (N (%)) | 524 (28) | 320 (39) | <0.01 | 553 (27) | 354 (37) | <0.01 |
| Tobacco exposure at home/work (N (%)) | 1048 (55) | 563 (69) | <0.01 | 1014 (49) | 548 (58) | <0.01 |
| Physically active (N (%)) | 901 (58) | 390 (53) | 0.05 | 969 (58) | 436 (52) | <0.01 |
